# Interplay of Charge Transfer and Local Triplet States in Donor–Acceptor-Based TADF Compounds

**DOI:** 10.1021/acs.jpclett.5c00241

**Published:** 2025-03-18

**Authors:** Tomas Serevičius, Sigitas Tumkevičius, Jelena Dodonova-Vaitku̅nienė, Saulius Juršėnas

**Affiliations:** † Vilnius University, Faculty of Physics, Institute of Photonics and Nanotechnology, Sauletekio 3, LT-10257 Vilnius, Lithuania; ‡ Vilnius University, Faculty of Chemistry, Institute of Chemistry, Naugarduko 24, LT-03225 Vilnius, Lithuania

## Abstract

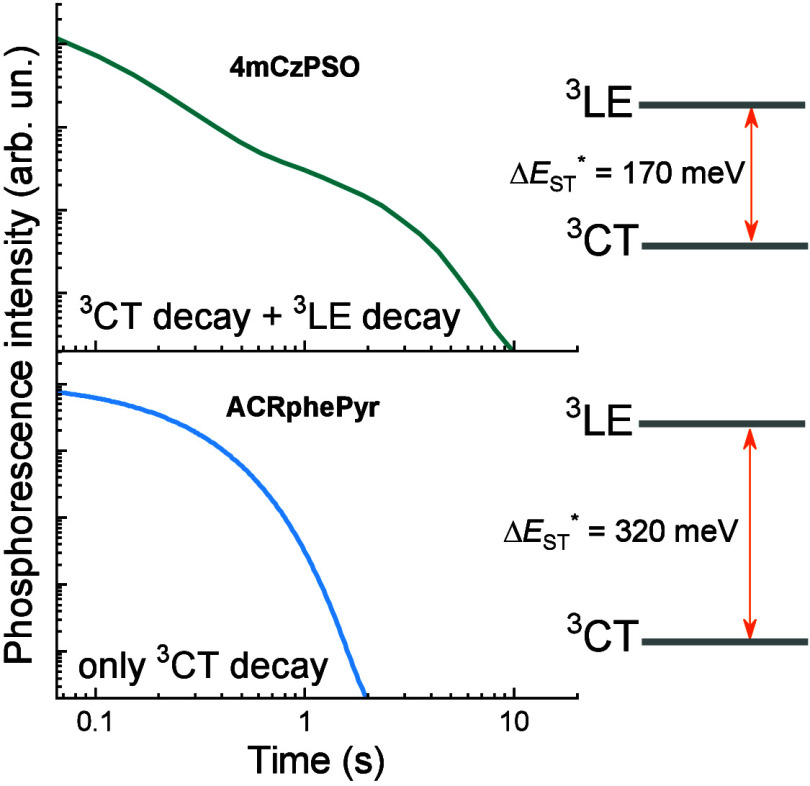

Donor–acceptor-based thermally activated delayed fluorescence (TADF) emitters have a complicated energy level scheme with multiple singlet and triplet energy levels. Among them, TADF compounds with the lowest-energy charge-transfer triplet state (type III compounds) are especially interesting due to their specific emission properties. We show that type III TADF compounds may show dual phosphorescence at low temperatures as well as weak high-energy emission at room temperature, somewhat resembling the phosphorescence spectrum of the local triplet. Such untypical behavior was demonstrated only in compounds with a low energy gap between the triplet states of different character, imposing efficient communication between them through vibronic coupling.

Decreasing the singlet–triplet energy gap of organic emitters (Δ*E*_st_) to negligible values is an elegant way to employ the dark triplet states in recombination through thermal activation and achieve near-unity internal electroluminescence quantum efficiency.^1,2^ Spatial separation of the highest-energy occupied (HOMO) and the lowest-energy unoccupied (LUMO) molecular orbitals is mandatory for a low Δ*E*_st_, typically achieved by constructing molecular emitters from electron-donating (D) and -accepting (A) units^3^ in a twisted orientation. Such thermally activated delayed fluorescence (TADF) compounds, having a decoupled HOMO and LUMO, possess complicated energy level schemes with multiple charge-transfer (CT) and local (LE) singlet and triplet energy states.^4^ Besides the need for a small gap between the lowest-energy singlet and triplet states (that is Δ*E*_st_), higher-energy triplet states must be involved.^5−8^ It was shown that reverse intersystem crossing (rISC) from T_1_ to S_1_ cannot be driven solely by spin–orbit coupling (SOC), and second-order coupling with T_2_ should be included. In this case, rISC is described as a two-step process. First, an equilibrium between T_1_ and T_2_ (of a CT (^3^CT) or a LE (^3^LE) nature) is formed through efficient vibronic coupling, followed by SOC between nearly degenerate ^1^CT and ^3^CT involving ^3^LE as the intermediate. Ideally, all ^1^CT and ^3^LE/^3^CT states should be nearly isoenergetic, maximizing the rISC.^6,9^

The coexistence of energetically close multiple singlet and triplet states in TADF compounds complicates the recombination processes. Simultaneous TADF and room-temperature phosphorescence may be observed,^10−12^ as well as dual phosphorescence (PH) from donor and acceptor units.^13^ In line with that, simultaneous phosphorescence of ^3^CT and ^3^LE was also reported in several TADF compounds.^14−16^ In this case, the authors suggested that the energy dispersion of the ^3^CT states would enable such dual phosphorescence. Previously, ^1^CT states were shown to suffer from the solid-state conformational disorder, leading to temporal emission peak instability and the subsequent decrease of rISC rates.^17,18^ Such behavior is a consequence of the specific molecular structure of TADF compounds, where the molecular body is made of singly bonded D and A units with evident rotational flexibility. When such molecules are placed in a solid surrounding, their D–A orientation (and the HOMO–LUMO overlap) may vary from molecule to molecule. In this way, every molecule is described with its unique ^1^CT energy, rISC, and radiative decay rate, leading to a potential decrease in the average decay rate when this conformational distribution is evident. Therefore, the same D–A angular distribution was suggested to also play an important role in ^3^CT emission. It was shown that the energy distribution of ^3^CT states is even larger than that of ^1^CT emission, leading to dual ^3^CT and ^3^LE phosphorescence. Initially, ^3^CT is observed from conformer states with a ^3^CT energy larger than that of ^3^LE. This ^3^CT emission competes with intramolecular triplet energy transfer from ^3^CT to ^3^LE, enabling dual phosphorescence. As some conformers were shown to have a ^3^CT energy lower than that of ^3^LE, only ^3^CT emission was observed in that case.

In this work, we present a photophysical analysis of two TADF compounds with ^3^CT as the lowest triplet state and different rotational flexibilities of the donor–acceptor angle. Although the singlet states showed typical ^1^CT energy dispersion, we found no evident conformational distribution of the ^3^CT states. On the other hand, dual low-temperature phosphorescence of ^3^CT and ^3^LE states was found for compounds with a smaller ^3^CT–^3^LE energy gap, imposing stronger vibronic coupling needed for efficient upconversion. As the thermal energy at 10 K is limited, phosphorescence from a higher-energy ^3^LE state may compete with slow TADF and internal conversion back to ^3^CT. Interestingly, long-lived high-energy decay following TADF was observed at room temperature, suspiciously reassembling low-temperature ^3^LE emission. Our results provide an alternative mechanism for dual ^3^CT and ^3^LE phosphorescence in TADF compounds and more insights into the photophysics of type III TADF compounds.

Two TADF compounds with different molecular structures and emission properties were selected (see Figure 1a). Compound **4mCzPSO**(19) is constructed from a tetramethylcarbazole (4mCz) donor and a pyrimidine-based acceptor unit. Four methyl units in a 4mCz fragment play an especially important role, remarkably increasing the steric hindrance between the D and A units and decreasing the twisting lability of the donor unit.^18^ Such a rigid molecular geometry of **4mCzPSO** leads to low conformational disorder.^19^ As a comparison, compound **ACRPhenPYR**(20) was selected. It has a 9,10-dihydroacridine donor unit with lower steric hindrance than 4mCz, leading to an enhanced conformational distribution of the D–A orientation.^20^ The emission data of **4mCzPSO** and **ACRPhenPYR** are shown in Table S1.

**Figure 1:**
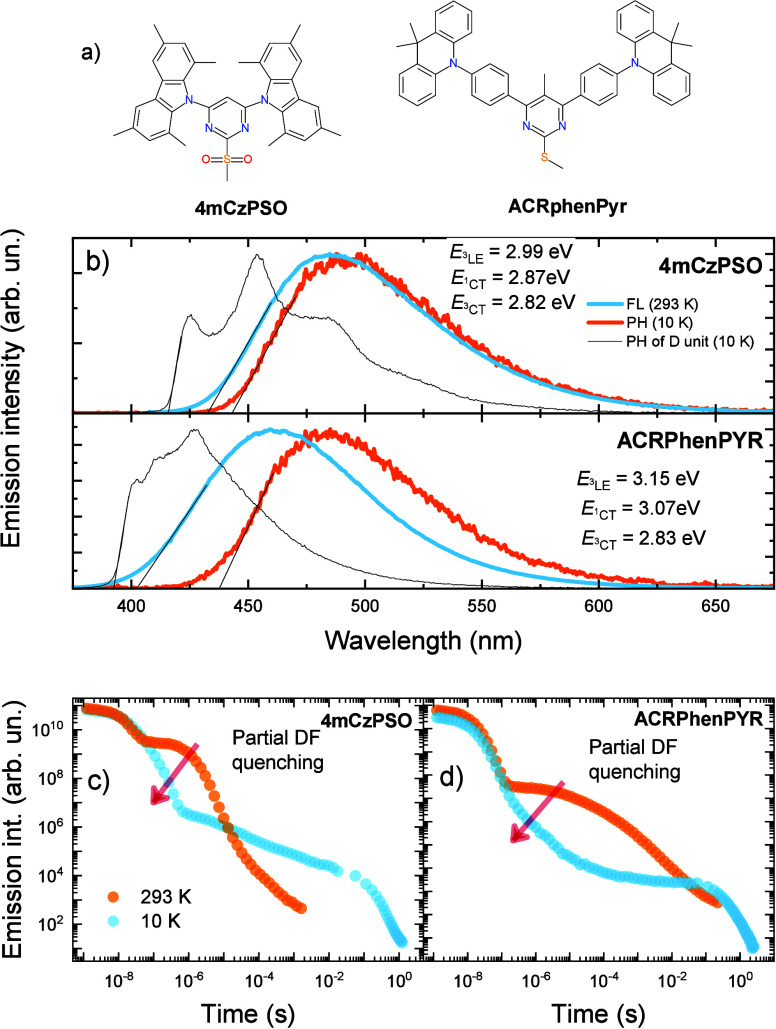
(a) Molecular structures of **4mCzPSO** and **ACRPhenPYR**. (b) Fluorescence (293 K, sky blue lines) and phosphorescence (10 K, orange lines) spectra of 1 wt % PMMA films of **4mCzPSO** and **ACRPhenPYR** and phosphorescence spectra of donor units. Numbers indicate the onset energies. Emission decay transients of 1 wt % PMMA films of (c) **4mCzPSO** and (d) **ACRPhenPYR** at room temperature (orange) and 10 K (sky blue).

Emission spectra of 1 wt % PMMA films of **4mCzPSO** and **ACRPhenPYR** are shown in Figure 1b. Both TADF compounds showed structureless fluorescence spectra of the charge-transfer type, typical for TADF compounds (onset energies of 2.87 eV (**4mCzPSO**) and 3.07 eV (**ACRPhenPYR**)). As observed for singlets, the lowest-energy phosphorescence spectra were also broad, without any trace of a vibronic pattern, implying a charge-transfer nature (onset energies of 2.87 and 3.07 eV for **4mCzPSO** and **ACRPhenPYR**, respectively). The energetically nearest local triplet states, residing at the donor units,^19,20^ were found to lie 170 and 320 meV above ^3^CT for **4mCzPSO** and **ACRPhenPYR**, respectively (Δ*E*_ST_*). The energy gap between ^1^CT and ^3^LE (Δ*E*_ST_) was somewhat smaller, 120 meV (**4mCzPSO**) and 80 meV (**ACRPhenPYR**). Therefore, both compounds can be called type III TADF emitters (the lowest-energy triplet is of a charge-transfer nature (see Figure S1)). As one of the possible proofs, fluorescence decay transients were measured at room temperature and 10 K (see panels c and d of Figure 1 and Figure S2). As we can see, evident delayed fluorescence can be seen at room temperature for both compounds (orange dots). However, an apparent portion of TADF can still be observed at low temperatures despite negligible thermal energy at 10 K and an evident potential barrier for triplet upconversion (see Figure S2 for more details). Such behavior, however, is in line with the dynamic two-step rISC model,^5−8,21^ where the initial step, population transfer from T_1_ to T_2_, is not activated by temperature and the population of T_2_ is non-zero even at 0 K.^7^ Then, type III TADF compounds with a sufficiently low Δ*E*_ST_ (as **4mCzPSO** and **ACRPhenPYR**) may show a non-zero rISC rate even at 10 K.^21^ Evidently, the TADF share at 10 K is lower for **ACRPhenPYR** with a larger Δ*E*_ST_*.

Further analysis of the low-temperature emission of **4mCzPSO** and **ACRPhenPYR** films was performed by investigating time-resolved fluorescence data (see Figure 2). Emission decay transients of **4mCzPSO** and **ACRPhenPYR** are shown in panels a and b, respectively, of Figure 2. Two excitation wavelengths were used (see Figure S3): a short wavelength for excitation to the donor unit (300 nm, blue dots) and a longer one for the direct excitation of CT states (405 and 355 nm (orange line) for **4mCzPSO** and **ACRPhenPYR**, respectively). Direct excitation to the absorption band of CT states should reduce the degree of involvement of emission from the donor unit and allow for the sole analysis of ^3^CT emission. **4mCzPSO** and **ACRPhenPYR** showed different behaviors of time-resolved emission decay. For **ACRPhenPYR**, the line shape of the fluorescence decay transient was the same for both excitation wavelengths. The decay of the ^3^CT state was single-exponential, with a time constant of 0.17 s. No evident temporal shifts of ^3^CT emission were observed in the time range of 0.22 ms to 0.62 s (see Figure 2h–j), when the emission peaked at the same energy as in steady-state spectra. For singlets, the typical initial red-shift was observed during the first ∼170 ns (see Figure 2g,h), amounting to 340 meV and indicating rather strong conformational disorder in weakly sterically restricted **ACRPhenPYR**. In the case of the remaining TADF, it was difficult to estimate whether any temporal shifts were present due to the low emission intensity (see Figure S4).

**Figure 2:**
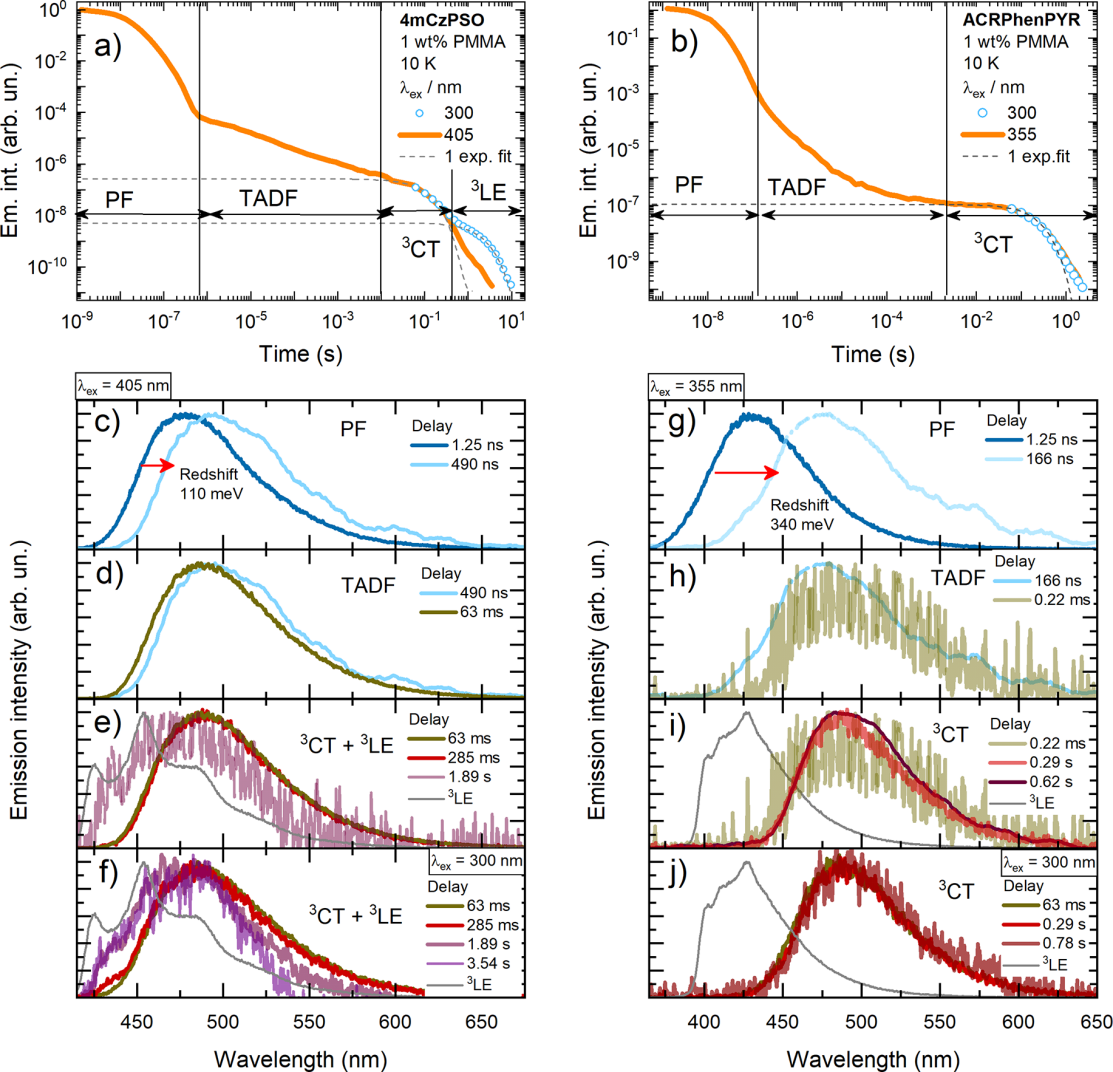
Emission decay transients of 1 wt % PMMA films of (a) **4mCzPSO** and (b) **ACRPhenPYR** at 10 K after excitation to the CT absorption band (orange lines) and the LE absorption band (sky blue dots). Time-resolved fluorescence spectra of 1 wt % PMMA films of (c–f) **4mCzPSO** and (g–j) **ACRPhenPYR** at different delay times and for different excitation wavelengths.

A completely different situation was observed for **4mCzPSO**. Single-exponential ^3^CT emission was also observed up to about 0.4 s after direct excitation to ^1^CT absorption (time constant of 0.085 s), followed by an additional weak emission decay tail. As opposed to **ACRPhenPYR**, the intensity of this low-intensity emission was remarkably enhanced when 300 nm laser impulses were used, enabling the excitation of individual acceptor fragments (blue dots in Figure 2a). This long-lived emission showed a single-exponential decay with an emission lifetime of 1.5 s. As we determined for **ACRPhenPYR**, no evident temporal shifts of ^3^CT phosphorescence were observed in the 60 ms to 0.4 s time interval. At later delays, additional high-energy emission emerged (see Figure 2e) and its intensity was remarkably higher when 300 nm excitation was used (see Figure 2f). The spectral line shape of this high-energy emission somewhat reassembles that of ^3^LE emission (see also Figure S5). Similar behavior was also observed for other type III TADF compounds,^14−16^ though no conformational distribution of ^3^CT states was found in our case. Moreover, dual phosphorescence was observed only in **4mCzPSO**, with a more sterically restricted molecular structure, which is less prone to conformational effects. Clearly, the conformational distribution of the ^3^CT states cannot explain the dual phosphorescence in **4mCzPSO**.

As an alternative possible pathway, a mechanism involving a dynamic rISC model^5−8^ was suggested. The energy level scheme of **4mCzPSO** and **ACRPhenPYR**, accounting for states involved in the phosphorescence decay, is shown in Figure 3. Initial steps (number 1 in Figure 3) are different under short- and long-wavelength excitations. In the first case, ^3^LE is populated after ISC from S_1_, followed by internal conversion (IC) to ^3^CT (number 2). In the latter case, direct absorption to ^1^CT results in the population of ^3^CT by ISC, probably involving intermediate triplet states.^8^ Despite the different excited-state deactivation trajectories, dual phosphorescence still was observed in both cases for **4mCzPSO**. It turns out that both ^3^CT and ^3^LE states are communicating. As discussed previously,^5^ the first step in rISC act is reverse internal conversion (rIC) from T_1_ to T_2_, mediated by vibronic coupling:
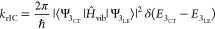
1

**Figure 3:**
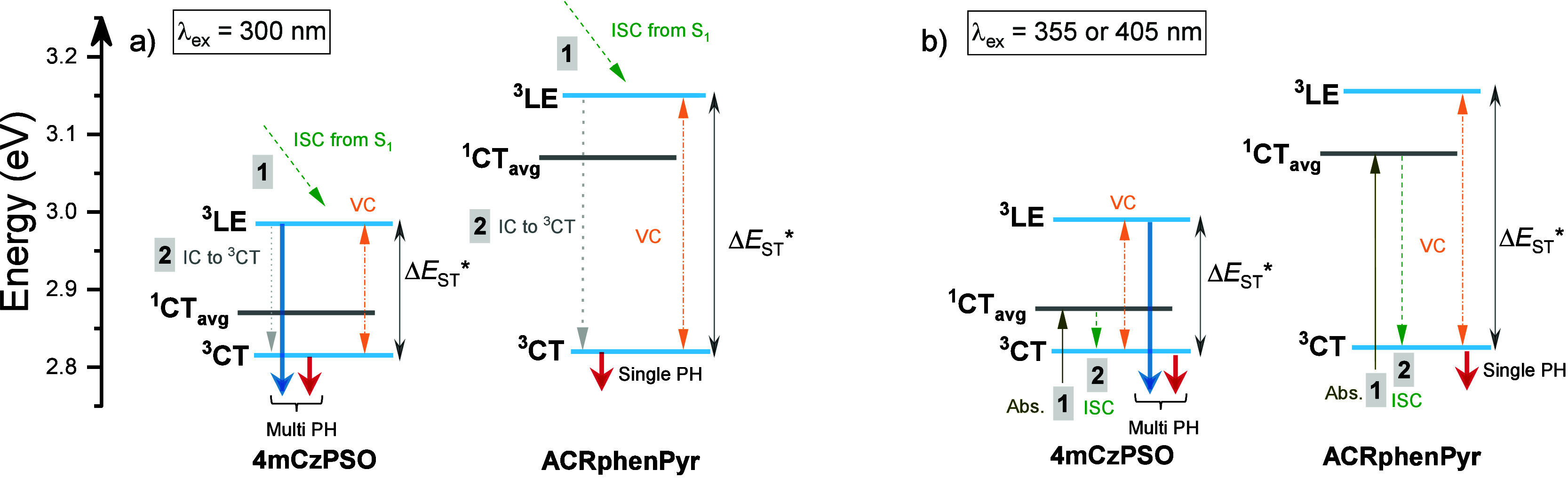
Energy level scheme and recombination pathways in (a) **4mCzPSO** and (b) **ACRPhenPYR** for different excitation wavelengths.

As we can see, the rate of rIC depends on the energy gap between the ^3^CT and ^3^LE states (*k*_rIC_ ∼ exp(Δ*E*_ST_*))^5^ and the strength of vibronic coupling (VC), which in TADF systems often originates from low-energy D–A rotations.^5,22^ In our case, Δ*E*_ST_* is evidently lower for **4mCzPSO** (170 meV vs 320 meV), though the D–A pair is less sterically restricted in **ACRPhenPYR**. Therefore, a substantial population of the ^3^LE state in **4mCzPSO** may be explained by more rapid rIC due to the lower Δ*E*_ST_*. It is also evident from the remarkably lower TADF share in **ACRPhenPYR** at 10 K (see Figure 2). As the lifetime of ^3^LE is substantial (about 1.5 s) and the rISC rate is rather low at 10 K, some of the ^3^LE population in **4mCzPSO** could undergo radiative decay to S_0_, as shown in Figure 2. However, it is clear that the ^3^LE emission is evidently enhanced when high-energy excitation is used. In this case, the population of ^3^LE clearly is larger after rapid ISC from S_1_ of the donor unit, and a larger share of LE triplets may undergo direct recombination to S_0_. For **ACRPhenPYR**, no ^3^LE emission was observed even for the high-energy excitation case, which is typical behavior for organic compounds. **ACRPhenPYR** has a larger S_1_–T_1_ energy gap as well as different energy level scheme, possibly leading to different SOC rates and then ^3^LE decay rates,^8,23^ which then may be outcompeted by more rapid rISC or nonradiative decay rates. To employ the delicate tuning of CT energy levels of **4mCzPSO** and **ACRPhenPYR**, additional doping of PMMA films with camphoric anhydride (CA) was used^24^ (see Figure S6 and Table S1). CA is a small molecule dopant with a very large ground-state dipole moment and can achieve an ultrafast reorientation in the pores of PMMA, increasing the polarity of the film. Both ^1^CT and ^3^CT showed typical positive solvatochromic behavior when the emission peak shifted up to 60 meV when 20 wt % CA was added (the dielectric constant of PMMA increased from 3.41 to 8.31). As the ^3^LE state is insensitive to polarity changes, a minor increase in the ^3^LE–^3^CT energy gap was observed for both compounds (from 170 to 320 meV and from 185 to 348 meV for **4mCzPSO** and **ACRPhenPYR**, respectively). Although this increase in Δ*E*_ST_* was only about 10% for **4mCzPSO**, it decreased the intensity of ^3^LE emission at 10 K, in line with our proposed model. In the case of **ACRPhenPYR**, no evident ^3^LE emission was observed, with or without additional CA doping.

In addition to the untypical emission properties of a 1 wt % PMMA film of **4mCzPSO** at 10 K, room-temperature emission was also unusual (see Figure 4). Parallel to the characteristic initial prompt and the later delayed fluorescence, a long-lived emission tail was observed (gray area in Figure 4a). Surprisingly, its intensity and lifetime were larger for shorter excitation wavelengths, especially for the case of direct excitation of the 4mCz unit (λ_ex_ ≥ 355 nm). More surprisingly, spectral onsets of this long-lived emission were of higher energy than those of PF and TADF, and its onset energy showed a blue-shift when the shorter excitation wavelength was used (see Figure 4b). The emission spectrum of this large-energy emission was obtained by subtracting DF spectra upon 420 nm excitation from that obtained at 300 nm (see Figure S7). Interestingly, the position of this DF spectrum quite well coincided with that of ^3^LE, despite the loss of vibronic structure, which may occur due to enhanced structural relaxation at room temperature. The existence of ^3^LE emission at room temperature, especially at energies larger than those of fluorescence, would be quite unexpected. However, the pronounced population of ^3^LE, evidenced by dual phosphorescence and its long lifetime, probably could enable the competing direct recombination pathway of ^3^LE to the ground state. In line with that, ^1^LE emission from the donor unit was also observed along the ^1^CT decay at short-wavelength excitation conditions (see Figure S8), nearly reassembling the fluorescence spectrum of the 4mCz unit. Therefore, an evident population of ^1^LE may lead to a pronounced population of ^3^LE through ISC and enable long-lived emission, as shown in Figure 4.

**Figure 4:**
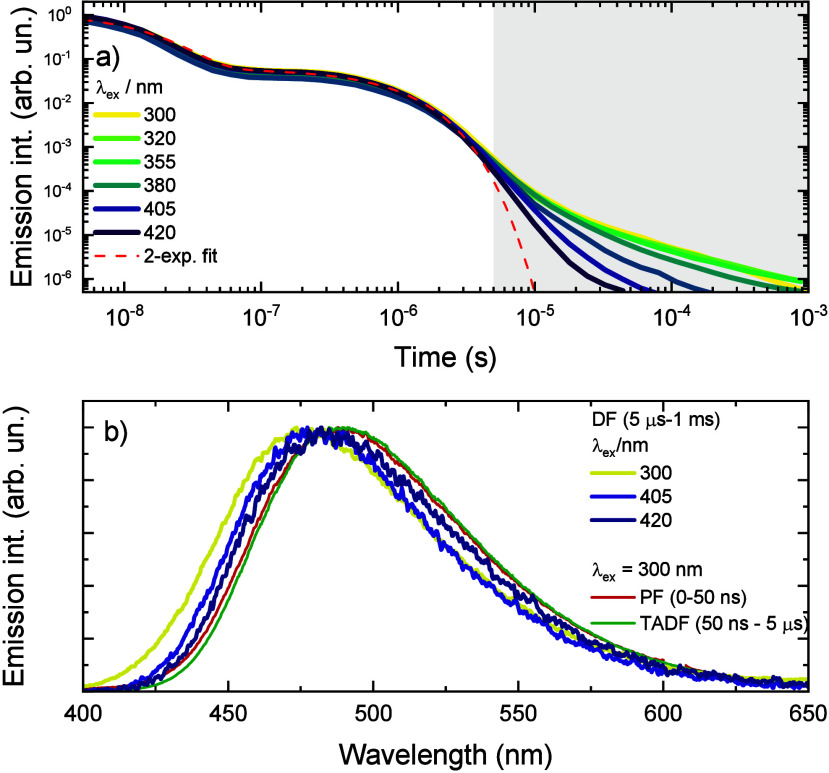
(a) Emission decay transients of a 1 wt % PMMA film of **4mCzPSO** for different excitation wavelengths at room temperature. (b) Prompt (PF, optical window of 0–50 ns), thermally activated delayed (TADF, optical window of 50 ns to 5 μs) fluorescence, and long-lived emission (DF, 5 μs to 1 ms) spectra of **4mCzPSO** at room temperature.

To conclude, we present a photophysical study of two type III TADF compounds, **4mCzPSO** and **ACRPhenPYR**, having different singlet–triplet energy gaps and structural rigidity. It was shown that decreasing the energy spacing between the ^3^LE and ^3^CT states (Δ*E*_ST_*) enables efficient coupling between them, leading to unusual emission properties. The dual phosphorescence of the ^3^LE and ^3^CT nature with evidently different lifetime was observed for **4mCzPSO** with a lower Δ*E*_ST_*. Interestingly, an apparent ^3^LE emission was obtained even after direct excitation to the charge-transfer absorption band, indicating strong communication between the states through vibronic coupling. Moreover, a long-lived high-energy emission band was observed for **4mCzPSO** also at room temperature with enhanced intensity after direct excitation of the donor unit (λ_ex_ ≥ 355 nm). Surprisingly, the position of this emission coincided with that of ^3^LE at 10 K, although the vibronic structure was absent. This again indicates the strong coupling between the triplet states for **4mCzPSO** (Δ*E*_ST_* = 170 meV). On the contrary, **ACRPhenPYR**, with an evidently larger Δ*E*_ST_* of 320 meV, showed typical emission features, such as excitation-independent emission spectra as well as single phosphorescence.

All in all, type III TADF compounds still show many new and intriguing effects that are worth examining in detail. Here we provide the possible mechanism of the unusual emission of **4mCzPSO** based on comprehensive experimental findings and hope this will foster further detailed studies of dual phosphorescence in TADF compounds.

## Experimental Methods

The photophysical properties were analyzed in 1 wt % PMMA (poly(methyl methacrylate)) films and 10^–5^ M toluene solutions (for absorption only). The solid-state samples were prepared by dissolving the compounds and polymer or host material in appropriate ratios in a toluene solution and then wet-casting the solutions on quartz substrates. The dielectric constant of the polymer host was altered by adding various amounts of (±)-camphoric anhydride^24^ (Fine Synthesis Ltd.). Time-integrated fluorescence, phosphorescence spectra, time-resolved fluorescence spectra, and fluorescence decay kinetics were recorded with a time-gated intensified iCCD camera (iStar DH340T, Andor) with a model SR-303i spectrograph (Shamrock) coupled with a nanosecond YAG:Nd^3+^ laser NT 242 with an optical parametric generator (Ekspla, pulse width of 5 ns, frequency of 4 Hz). Decay transients were obtained by exponentially increasing the delay and integration time.^25,26^ Fluorescence decay transients at 10 K were composed of two parts. The first part of the 1.25 ns to 250 ms time range was measured as described in refs (25) and (26). Since the phosphorescence lifetime is larger than the interval between laser pulses, the remaining PH from the last pulse was omitted. The second part (delay time of >250 ms) was obtained by irradiating samples with the same laser for 1 s to reach steady-state conditions and then measuring the PH emission with an external homemade shutter following the procedure from refs (25) and (26). All samples were mounted in a closed cycle He cryostat (Cryo Industries 204 N) for oxygen-free conditions.

## Supporting Information Available

Energy level scheme of TADF compounds, fluorescence decay transients in the range of 10–290 K, detailed absorption spectra, time-resolved fluorescence spectra at 10 K, deconstructed delayed emission spectra of **4mCzPSO** at 10 K, and detailed emission properties with additional CA doping (PDF)
