# Supplementary material for: Interplay of Charge Transfer and Local Triplet States in Donor–Acceptor-Based TADF Compounds
Source: J Phys Chem Lett. 2025 Mar 18;16(12):3100–5. doi: 10.1021/acs.jpclett.5c00241 (PMC11956135; doi:10.1021/acs.jpclett.5c00241)
Supplement: Supplementary file 1 — jz5c00241_si_001.pdf [file jz5c00241_si_001.pdf]

Supporting Information

Interplay of Charge-Transfer and Local Triplet States in Donor-Acceptor-Based TADF Compounds

Tomas Serevičius[a*], Sigitas Tumkevičius[b], Jelena Dodonova-Vaitkūnienė[b], Saulius Juršėnas[a]

[a] Vilnius University, Faculty of Physics, Institute of Photonics and Nanotechnology, Sauletekio 3, LT-10257 Vilnius, Lithuania.

[b] Vilnius University, Faculty of Chemistry, Institute of Chemistry, Naugarduko 24, LT-03225, Vilnius, Lithuania.

*Corresponding author: tomas.serevicius@tmi.vu.lt

Table 1 Emission data of **4mCzPSO** and **ACRPhenPYR** 1wt% PMMA. Spectral onsets were used to estimate emission energies. No delayed fluorescence lifetime (τ_{DF}) could be estimated for **ACRPhenPYR** due to the multiexponential temporal profile.

	E_{PL} (eV)	E_{3LE} (eV)	E_{3CT} (eV)	Φ_{PF}	Φ_{DF}	τ_{PF} (ns)	τ_{DF} (µs)
**4mCsPSO**	2.87	2.99	2.82	0.05	0.22	12	1
**ACRPhenPYR**	3.07	3.15	2.83	0.31	0.40	14	-*

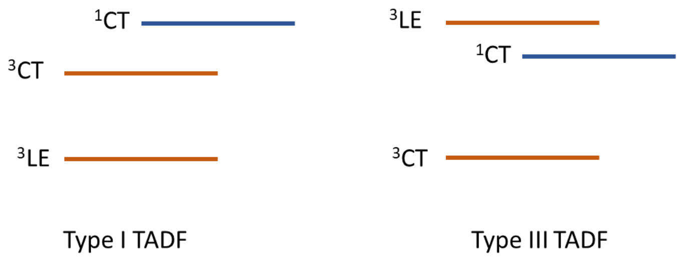

Fig. S1 Energy level scheme of Type I and Type III TADF compounds.

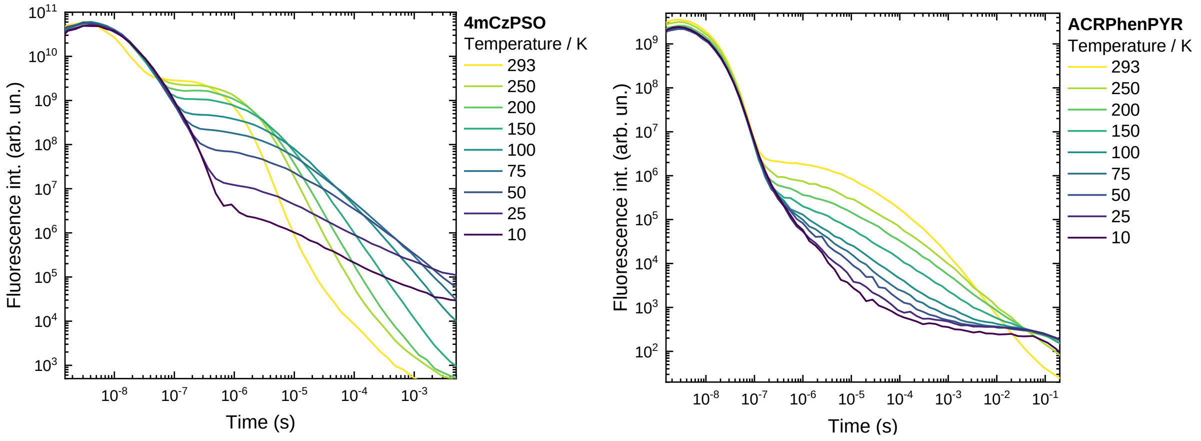

Fig. S2 Fluorescence decay transients of 1 wt% PMMA films of **4mCzPSO** (left) and **ACRPhenPYR** (right) at 10 – 293K temperatures.

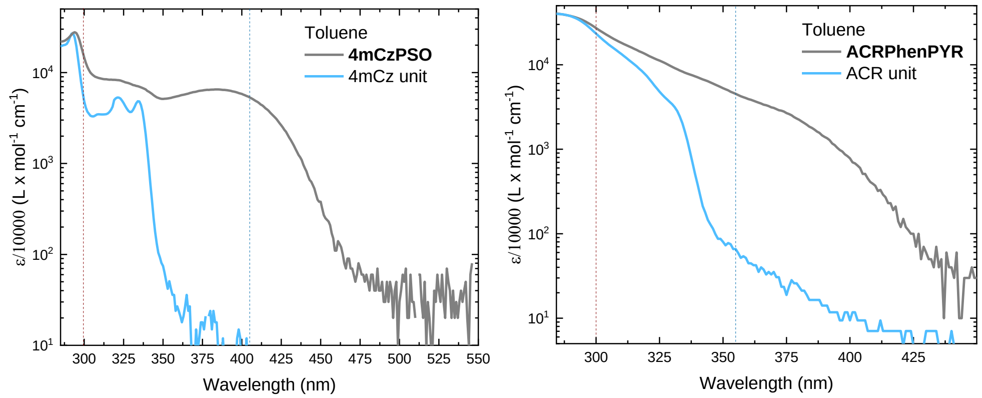

Fig. S3 Absorption spectra of **4mCzPSO**, 4mCz donor unit (left) and **ACRPhenPYR**, ACR donor unit (right) in 10^{-6} M toluene. Absorption spectra of donor units were vertically shifted for easier comparison. Dotted lines denote the laser wavelength used for transient measurements.

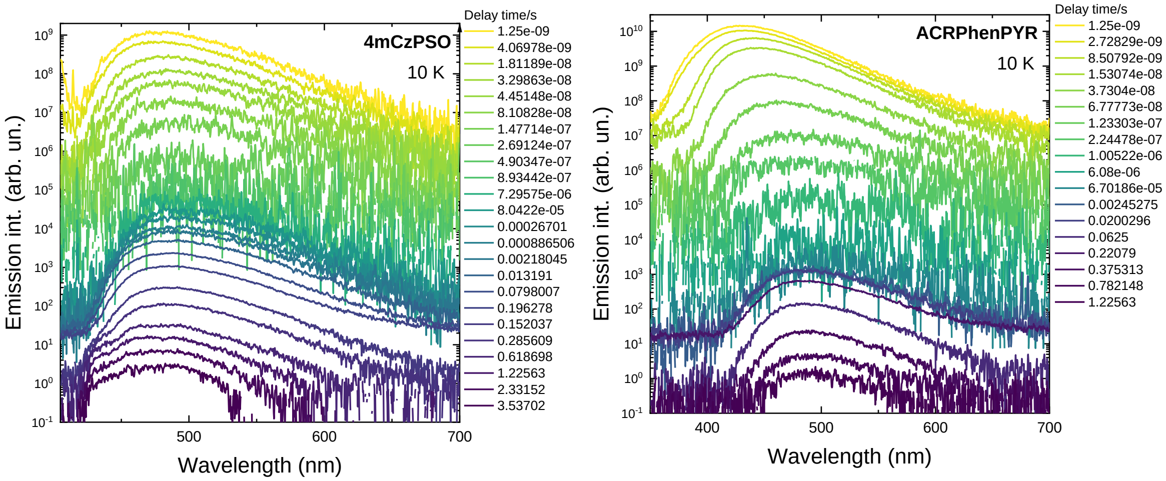

Fig. S4 Time-resolved emission spectra of 1 wt% PMMA films of **4mCzPSO** (left) and **ACRPhenPYR** (right) at 10 K. λ_{ex} was 405 nm for **4mCzPSO** and 355 nm for **ACRPhenPYR**.

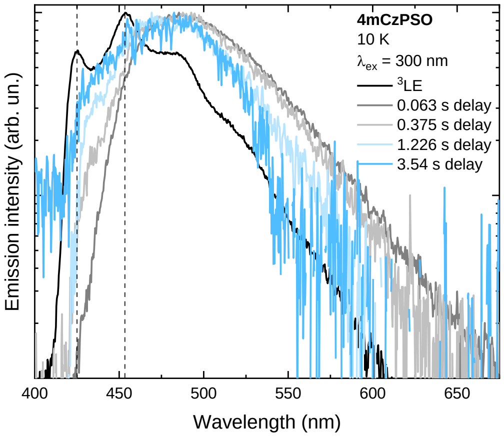

Fig. S5 Log-scaled normalized time-resolved emission spectra of 1 wt% PMMA films of **4mCzPSO** at 10 K at several delay times (color lines) and time-integrated ^{3}LE emission spectrum (black line).

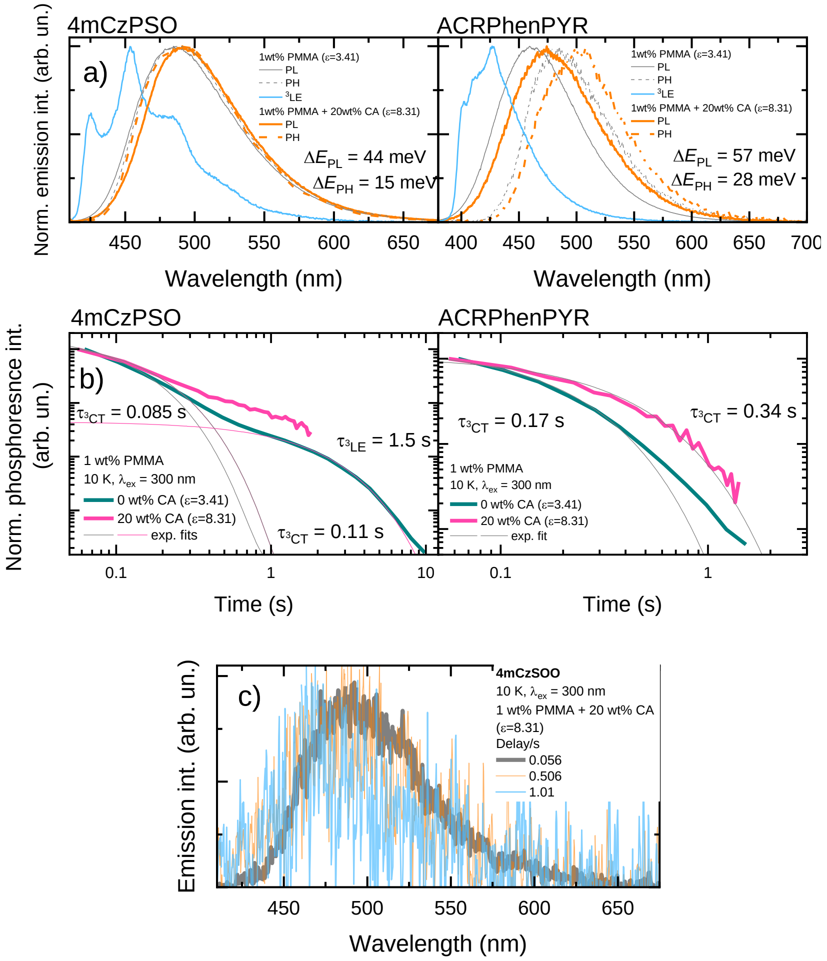

Fig. S6 a) Fluorescence (293 K), phosphorescence (10 K) spectra of 1wt% PMMA films of **4mCzPSO** and **ACRPhenPYR** and phosphorescence spectra of donor units (thin lines). Thick lines show fluorescence and phosphorescence spectra when additional 20wt% doping with camphoric anhydride (CA) was used. b) Phosphorescence decay transients of 1wt% PMMA films of **4mCzPSO** and **ACRPhenPYR** at 10 K after excitation to CT absorption band (green lines) and phosphorescence decay transients at the same conditions when additional 20wt% doping with CA was used (dark red lines). Thin lines are exponential fits of ^{3}CT (grey lines) and ^{3}LE decay (red line). c) Time-resolved fluorescence spectra of 1wt% PMMA film of **4mCzPSO** with additional 20wt% CA doping at 10 K.

Table S2 Energy gaps between different singlet and triplet states of 1 wt% PMMA films of **4mCzPSO** and **ACRPhenPYR** with and without the additional doping with camphoric anhydride.

	1 wt% PMMA (ε = 3.41)			1 wt% PMMA + 20 wt% CA (ε = 8.31)		
	ΔE_{1CT3LE} (meV)	ΔE_{1CT3CT} (meV)	ΔE_{3CT3LE} (meV)	ΔE_{1CT3LE} (meV)	ΔE_{1CT3CT} (meV)	ΔE_{3CT3LE} (meV)
**4mCsPSO**	120	50	170	186	~0	185
**ACRPhenPYR**	80	240	320	137	211	348

To alter the polarity of PMMA film, additional doping with camphoric anhydride (CA) was used[1–3]. This increased the film's dielectric constant from 3.41 at 0 wt% load to 8.31 when 20 wt% of CA was added. Typical positive solvatochromic shifts were observed for both fluorescence and phosphorescence (though singlets were more susceptible to polarity changes). Both ΔE_{ST} and ΔE_{ST}^* were larger at 20wt% CA doping load due to the more stabilized ^{3}CT, though the gap between ^{1}CT and ^{3}CT decreased, especially for **4mCzPSO.** Very weak ^{3}LE emission was observed for **4mCzPSO** for 300 nm excitation in doped PMMA film (only weak shoulder around 450 nm at 0.5-1s delay, see Fig. S6 c), contrary to the undoped PMMA where the intensity of ^{3}LE was significant. Larger energy splitting between ^{3}CT and ^{3}LE energy levels lowered the population of ^{3}LE, leading to diminished ^{3}LE decay. Similarly, as for ^{1}CT emission, the lifetime of ^{3}CT was a bit larger in the more polar surrounding.

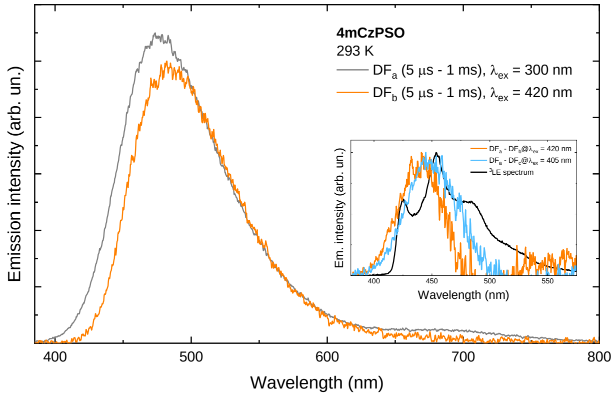

Fig. S7 Delayed emission (optical window 5 µs – 1 ms) spectra of **4mCzPSO** at 293 K after excitation with 300 nm (DF_a, grey line) and 420 nm (DF_b, orange line) light. The grey spectrum was vertically shifted to reassemble the low-energy shoulder of the orange spectrum. The inset shows the spectral difference of DF_a – DF_b (orange line) and the DF_a – DF_c (sky-blue line), where the DF_c is delayed emission spectrum of **4mCzPSO** after 405 nm excitation. In the latter case, DF_a was vertically shifted to match DF_c.

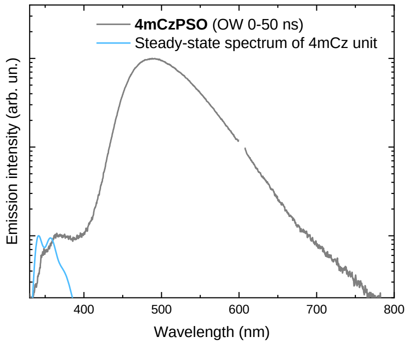

Fig. S8 Fluorescence spectra (optical window 0 ns – 50 ns, λ_{ex} = 300 nm) of 1 wt% PMMA film of **4mCzPSO** at 293 K (grey line) and of 4mCz unit (vertically shifted, sky-blue line).

References

(1) Cotts, B. L.; McCarthy, D. G.; Noriega, R.; Penwell, S. B.; Delor, M.; Devore, D. D.; Mukhopadhyay, S.; De Vries, T. S.; Ginsberg, N. S. Tuning Thermally Activated Delayed Fluorescence Emitter Photophysics through Solvation in the Solid State. *ACS Energy Lett.* **2017**, *2* (7), 1526–1533. https://doi.org/10.1021/acsenergylett.7b00268.

(2) Delor, M.; McCarthy, D. G.; Cotts, B. L.; Roberts, T. D.; Noriega, R.; Devore, D. D.; Mukhopadhyay, S.; De Vries, T. S.; Ginsberg, N. S. Resolving and Controlling Photoinduced Ultrafast Solvation in the Solid State. *J. Phys. Chem. Lett.* **2017**, *8* (17), 4183–4190. https://doi.org/10.1021/acs.jpclett.7b01689.

(3) Serevičius, T.; Skaisgiris, R.; Dodonova, J.; Fiodorova, I.; Genevičius, K.; Tumkevičius, S.; Kazlauskas, K.; Juršėnas, S. Temporal Dynamics of Solid-State Thermally Activated Delayed Fluorescence: Disorder or Ultraslow Solvation? *J. Phys. Chem. Lett.* **2022**, *13* (7), 1839–1844. https://doi.org/10.1021/acs.jpclett.1c03810.
